# Evolutionary study of potentially zoonotic hepatitis E virus genotype 3 from swine in Northeast Brazil

**DOI:** 10.1590/0074-02760180585

**Published:** 2019-06-03

**Authors:** Edmilson Ferreira de Oliveira-Filho, Debora RL dos Santos, Ricardo Durães-Carvalho, Adalúcia da Silva, Gustavo Barbosa de Lima, Antônio Fernando B Batista, Lindomar J Pena, Laura HVG Gil

**Affiliations:** 1 Fundação Oswaldo Cruz-Fiocruz, Instituto Aggeu Magalhães, Departamento de Virologia, Recife, PE, Brasil; 2 Charité-Universitätsmedizin Berlin, Corporate Member of Freie Universität Berlin, Humboldt-Universität zu Berlin, and Berlin Institute of Health, Institute of Virology, Berlin, Germany; 3 Universidade Federal Rural do Rio de Janeiro, Instituto de Veterinária, Seropédica, RJ, Brasil; 4 Fundação Oswaldo Cruz-Fiocruz, Instituto Aggeu Magalhães, Departamento de Microbiologia, Recife, PE, Brasil; 5 Universidade Federal Rural de Pernambuco, Departamento de Medicina Veterinária, Recife, PE, Brasil

**Keywords:** zoonotic hepatitis E virus, swine, genotype 3

## Abstract

Hepatitis E virus (HEV), an emerging virus associated with acute hepatic disease, leads to thousands of deaths worldwide. HEV has already been reported in Brazil; however, there is a lack of epidemiological and molecular information on the genetic variability, taxonomy, and evolution of HEV. It is thus unclear whether hepatitis E is a neglected disease in Brazil or it has low relevance for public health in this country. Here, for the first time, we report the presence of HEV in Northeast Brazil. A total of 119 swine faecal samples were screened for the presence of HEV RNA using real-time polymerase chain reaction (RT-PCR) and further confirmed by conventional RT-PCR; among these, two samples were identified as positive. Molecular evolution analyses based on capsid sequences revealed that the samples had close proximities to HEV sequences belonging to genotype 3 and were genetically related to subtype 3f isolated in humans. Parsimony ancestral states analysis indicated gene flow events from HEV cross-species infection, suggesting an important role of pig hosts in viral spillover. HEV’s ability for zoonotic transmission by inter-species host switching as well as its possible adaptation to new animal species remain important issues for human health.

Hepatitis E is an emerging zoonotic disease caused by the hepatitis E virus (HEV). HEV is a member of the genus *Orthohepevirus* A and can be divided into eight major genotypes^1^ and 43 subtypes (assigned and unassigned).^2^ Among them, only HEV-3 and HEV-4 have been reported in humans and animals worldwide, and they are known to cause cross-species infection, posing a threat of zoonotic transmission.^3^ In Brazil, HEV-3 has been detected in humans and in swine in the South, Southeast, and Central-West regions.^4^ ^,^ ^5^ ^,^ ^6^ ^,^ ^7^ ^,^ ^8^ To date, HEV isolates have not been detected in Northeast Brazil. Swine production in this area is characterised by a low technical level and by poor investments in mechanisation, technology, and biosecurity practices. In this study, we screened for and performed phylogenetic analyses based on different genome segments of HEV strains circulating among domestic swine in Northeast Brazil.

A total of 119 faecal samples were individually collected in 2017 from two to six-month-old animals from eight farms using both intensive and extensive production systems located within a high HEV seroprevalence area.^9^ Viral RNA was extracted from a 200 µL faecal suspension using the ReliaPrep™ Viral Total Nucleic Acid Purification Kit (Promega, Brazil) and screened by a previously described real-time polymerase chain reaction (RT-PCR) method^10^ using the NextGeneration ECO One-Step HotStart RT-qPCR Kit (DNA Express Biotecnologia, Brazil). Conventional HEV RT-PCR, using a set of previously designed primers to amplify the ORF2 region,^11^ ^,^ ^12^ was used to confirm the positive samples. Primers for sequencing the complete capsid region were designed based on the pertinent literature^13^ ^,^ ^14^ and on an alignment using 156 partial genomic Brazilian and HEV-3 subtype reference sequences (Tables I-II). RT-PCR was performed using the SuperScript™ III One-Step RT-PCR System with Platinum™ Taq DNA Polymerase (Invitrogen, USA) according to manufacturer’s instructions. Nested PCR was performed using PCR Master Mix 2X (Promega, Brazil), and the obtained PCR products were purified with the QIAquick Gel Extraction Kit^TM^ (Qiagen) before being sent for sequencing.

Initially, sequences were aligned using MUSCLE v3.6 software.^15^ Markov models of nucleotide substitution were chosen using jMoldelTest v.2 software.^16^ Sequences were retrieved from GenBank, and the accession numbers are displayed on the phylogenetic trees. Evolutionary studies were conducted using the maximum likelihood (ML) and maximum parsimony (MP)^17^ inference methods implemented in FastTree v.2.1.7^18^ and Mesquite v.3.5.1^19^ software. ML analysis was conducted with the standard default GTR + CAT with 20 gamma distribution parameters and a mix of the nearest-neighbour interchanges (NNI) and sub-tree-prune-regraft (SPR). The MP method, in which the best phylogenetic tree is determined based on favouring the fewest evolutionary changes, was performed using the parsimony ancestral states algorithm with the SPR rearranger to trace the history of character evolution on 1.000 trees.^17^ The reliability of the tree nodes was obtained from Shimodaira-Hasegawa (SH-like) test support values with 1000 replications.^20^ Phylogenetic signals were assessed via likelihood mapping analysis using Tree-Puzzle v.5.2 software.^21^ ^,^ ^22^ The pairwise homoplasy index (PHI) for the Recombination search was implemented in SplitsTree v.4.14.6 following the default settings.^23^ Lastly, pairwise and patristic distances were inferred using MEGA v.7^24^ and PATRISTIC^25^ software, respectively.

**TABLE I:** Oligonucleotides used to amplify the hepatitis E virus (HEV) capsid region

Regions	Oligonucleotides	Obtained from	Genome position^*^
Cap1 Ext F	GCGCAGGTYTGTGTTGATGT	This study	4969 - 4988
Cap1 Ext R	TACTGGGCATRGTTRGAYGCCTC	This study	5702 - 5724
Cap1 Int F	GGGYTGGTRCATAACCTYATTGG	This study	5017 - 5039
Cap1 Int R	GCCATAATRTGTGTRTTGGTGCC	This study	5675 - 5697
Cap2 Ext F	TCACCGGCCCCYGAYAC	This study	5510 - 5526
Cap2 Ext R	ARSCGRTGGCGGGCTGT	This study	6146 - 6162
Cap2 Int F	TGCGACGACAGTATAAYYT	This study	5565 - 5583
Cap2 Int R	GTRTACCGRGATACACG	This study	6125 - 6141
Cap3 Ext F	TGGTGATGCTYTGYATTCATGG	This study	5994 - 6015
Cap3 Ext R	ACCARTCMAGAGARCGGG	This study	6702 - 6719
CAP3 Int F	CTTGAYTTYGCGYTAGARCTTGA	This study	6068 - 6089
Cap3 Int R	CCTGRGCCCCTGTTGCYA	This study	6678 - 6695
Cap4 Ext F	GAGTAYGAYCAGACTACGTATGG	This study	6605 - 6627
Cap4 Int F	TCCACCAACCCGATGTATGT	This study	6632 - 6651
15T-aTag	CCAACGACCGGGAGGCCATTTTTTTTTTTTTTTV	^(14)^	Poly-A tail
TAG	CCAACGACCGGGAGGCCA		-

*: based on GenBank accession code AF082843.

**TABLE II:** Sequences comparison among hepatitis E virus 3 (HEV-3) e-f-g subtypes extracted from the well-supported monophyletic clade highlighted in Fig. 1

	Accession numbers	Countries	Hosts	Collection dates	Subtypes	Pairwise distances	Patristic distances
1	AF455784	Kyrgyzstan	Swine	1987-89(2004)	3g	0.226	0.3739
2	JQ013795	France	Human	2006	3e^§^	0.215	0.3339
3	AB291958	Japan	Human	2004	3e	0.203	0.3607
4	AB780453	Japan	Wild boar	2011	3e	0.218	0.3757
5	AB248521	Japan	Swine	2006^*^	3e	0.188	0.3497
6	AB248522	Japan	Swine	2006	3e	0.187	0.3537
7	JQ026407	Japan	Monkey^§§^	2009	3e	0.211	0.3667
8	KP698919	Italy	Swine	2012	3e	0.206	0.3575
9	KF922359	France	Human	2009-10	3e	0.212	0.3691
10	JQ953665	France	Swine	2006	3e	0.214	0.3518
11	FJ998015	Germany	Wild boar	2007	3e	0.200	0.3505
12	HM055578	Hungary	Swine	2005	3e	0.202	0.3278
13	AB290313	Mongolia	Swine	2006	3	0.190	0.2762
14	EU723512	Spain	Swine	2009	3	0.189	0.2468
15	KJ873911	Germany	Human	2013	3	0.177	0.2695
16	KT581447	Sweden	Swine	2015^*^	3	0.184	0.2861
17	KT581444	Sweden	Swine	2015^*^	3	0.161	0.2740
18	KT581446	Sweden	Wild Boar	2015^*^	3	0.167	0.2387
19	EU360977	Sweden	Swine	2007^*^	3	0.175	0.2633
20	KT581443	Sweden	Swine	2015	3	0.205	0.2810
21	KT581445	Sweden	Wild boar	2015	3	0.174	0.2832
22	EU723516	Spain	Swine	2008^*^	3	0.134	0.1790
23	EU723514	Spain	Swine	2008^*^	3	0.141	0.1788
24	EU723513	Spain	Swine	2008^*^	3	0.136	0.1767
25	LC164712	Japan	Human	2007	3	0.142	0.1953
26	LC055973	Japan	Human	2008	3	0.149	0.1832
27	AB850879	Japan	Human	2012	3	0.140	0.1874
28	EU495148	France	Human	2008^*^	3f	0.139	0.1722
29	JN906975	France	Swine	2010	3f	0.111	0.1700
30	JN906974	France	Human	2010	3f	0.111	0.1700
31	JN906976	France	Swine	2010	3f	0.213	0.1700
32	KC166971	France	Human	2008	3f	0.114	0.1713
33	LC055972	Japan	Human	2012	3f	0.114	0.1827
34	KT581448	Spain	Swine	2015^*^	3f	0.118	0.1742
35	JQ953666	France	Swine	2008	3f	0.135	0.1499
36	FJ956757	Germany	Human	2005	3f	0.147	0.1899
37	KT591533	France	Human	2013	3f	0.124	0.1624
38	KT591532	France	Human	2013	3f	0.124	0.1624
39	KF891380	Italy	Swine	2013^*^	3f	0.131	0.1657
40	AB291961	Japan	Human	2004	3f	0.137	0.1810
41	EU375463	Thailand	Swine	2008^*^	3f	0.141	0.1752
42	AB369687	Japan^**^	Human	1998	3f	0.123	0.1657
43	FJ653660	Thailand	Human	2008	3f	0.148	0.1753
44	S26	Brazil	Swine	2017	3f	0.000	0.0000

HEV-3 subtypes references(2) are in bold. *: collection date was not available and GenBank deposition year is displayed; **: patient traveled to Thailand § classified as 3f on the GenBank §§ probably of human origin.

From a total of 119 samples, two samples (1.68%) originating from three- (S67) and five-month-old (S26) clinically healthy piglets yielded 304-nucleotide (nt)-long DNA fragments corresponding to the HEV capsid region (Accession numbers: MH664123 and MH664124). The positive animals were from two distinct farms [Supplementary data (Fig. 2)]. Evolutionary analysis showed that both the S26 and S67 HEV sequences clustered within genotype HEV-3 together with the subtype 3f derived from a patient with acute hepatitis E in Japan who is known to have travelled to Bangladesh (see AB369387) (Fig. 1A). Additionally, we performed further analyses on a 1503-nt-long capsid sequence fragment isolated from sample S26. Analysis of this larger fragment along with 96 HEV-3 representative reference sequences retrieved using Boolean terms [Hepatitis E virus(Organism)] AND ORF2(Gene name) from GenBank, 43 of which had been previously assigned to clade 3e-3f-3g, thus confirming S26 to be phylogenetically related to clade 3f (see Fig. 1C clade two highlighted by the dotted lines, SH-like support ≥ 80%). These data were extrapolated from the consistent monophyletic origin and asymmetric phylogenetic tree backbone support [Fig. 1B, Supplementary data (Fig. 1)]. Intriguingly, although the sequence exhibited a high divergence, as demonstrated by the length of the tree branch (red line), we observed a proximity to the data obtained from the pairwise and patristic distances of HEV isolated from humans (KT591533.1 and KT591532.1) (Fig. 1B, Table II). Further gene flow analysis of S26 showed an ancestry degree and viral gene stream among HEV-3 virus populations typically found in human and animal hosts, suggesting both a switch of host direction and that this HEV-3 subtype 3f strain is probably of zoonotic origin (Fig. 1C). Similar patterns were also observed among strains of the HEV-e-f-g monophyletic clade, revealing a continuous flow of viral gene interchange and spread among sequences from human and animal hosts.

**Figure:**
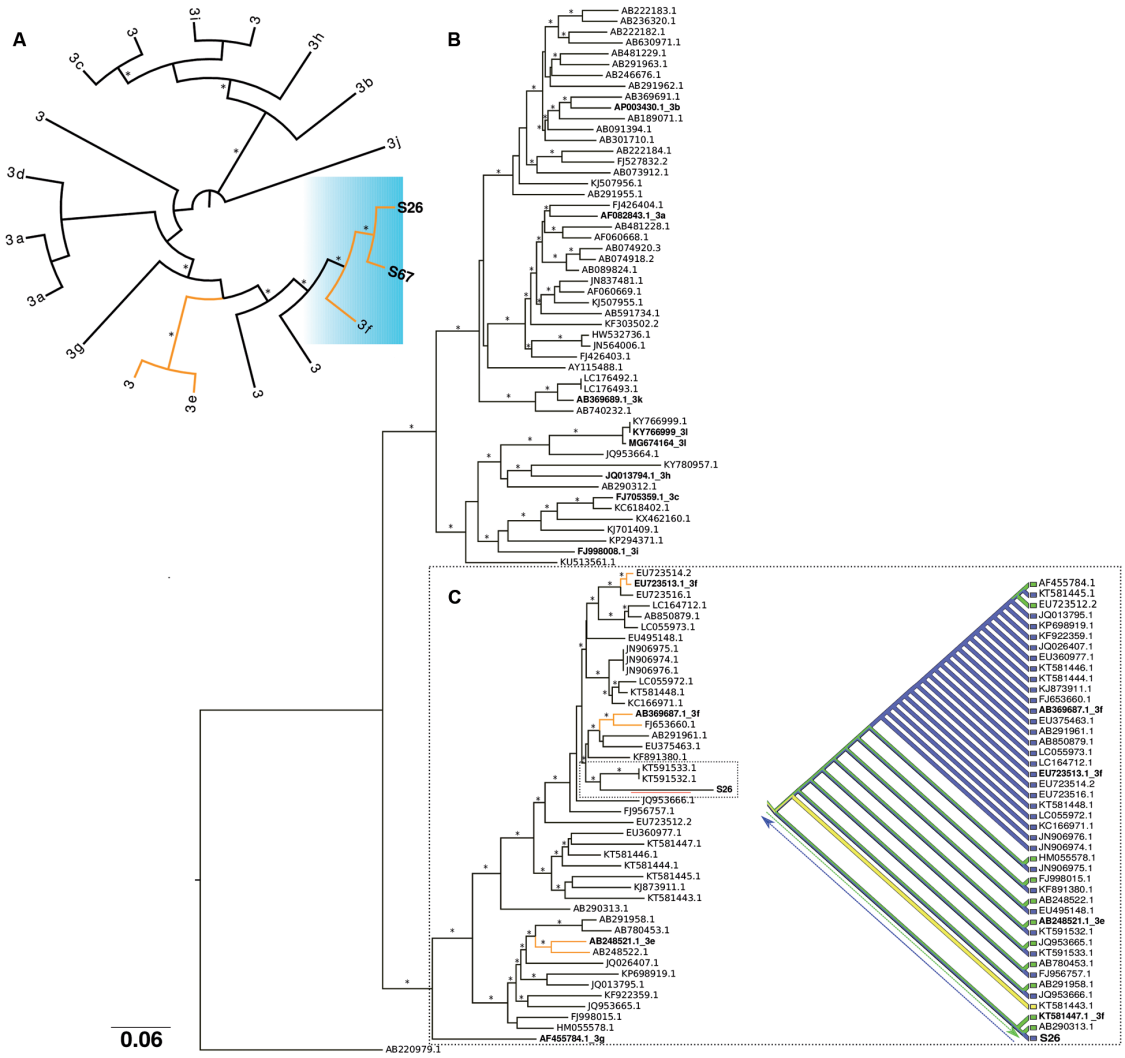
Phylogenetic analyses based on a 304 nt fragment (A) and 1503 nt (B and C) fragment of the hepatitis E virus 3 (HEV-3) capsid gene. Maximum likelihood (ML) phylogenetic tree showing the HEV samples in this study (blue gradient background: S26 and S69) clustering with subtype 3f isolated from humans (A). In (B), sequence S26 is in a rectangular dashed box. Subtype reference sequences^(2)^ are in bold. The red line under a tree branch highlights the sequence divergence according to the tree bar scale. The branches in orange colour highlight the HEV 3f and 3e clades. The asterisks along tree branches represent SH-like support values of ≥ 0.75 (B). Parsimony ancestral states analysis inferring the character history of the sequences extracted from the second large clade from (B). Each colour represents HEV-3 isolated from different hosts, countries, and different sampling years (see Table II). The dotted blue and green arrows show our hypothesis about the continuous flow showing viral gene interchange and spread among HEV isolated from different hosts (C).

Although a human virus was recently suggested to be the most likely ancestor of *Orthohepevirus* A,^26^ the human or animal origin of HEV remains under debate. In addition, with regard to its progressive host range expansion, it appears that host switching has played an important role during the evolutionary history of HEV and that genotype differences might have arisen via cyclical adaptation to different hosts.^27^ Although there is a clear host range distinction among HEV genotypes, HEV-3 and HEV-4 viruses appear to be circulating between animal and human hosts.

Our results are the first confirmation of HEV circulating among domestic swine farms in Northeast Brazil. The HEV RNA prevalence rate found here is markedly low considering the seroprevalence rates of up to 95% found in the same region.^9^ Nevertheless, our findings are similar to those obtained in other countries such as Germany, China, Japan, and India.^13^ ^,^ ^28^ ^,^ ^29^ ^,^ ^30^ Differences between serological and molecular prevalence rates are expected and likely to be related to animal age because HEV RNA is more easily detected in piglets that are two to six months old.^5^

Following the ICTV recommendation,^2^ S26 was assigned to subtype 3f, which has already been detected in Brazil.^7^ Although we were able to assign it successfully, a clear subtype separation based on pairwise or patristic distances was not possible (Table II). This divergence is likely related to differences in HEV-3 evolutionary rates among different subtypes, which seem to be higher among human-associated lineages.^27^ In addition, the marked heterogeneity observed within the subtype 3f and 3e-3f-3g clade might be an evolutionary hallmark, for example resulting from the accumulation of mutations positively selected through successful infection in different hosts.^27^ Thus, these results illustrate that subtyping inside this clade can be challenging and might indicate the possibility of the future emergence of new subtypes if additional new heterogenic sequences appear.

The close relationship between HEV strains isolated from different host species points towards interspecies transmission. In addition, the gene flow study corroborated a possible evolutionary host switch origin and zoonotic potential. Thus, the molecular evidence indicates that the HEV strains identified here are potentially zoonotic and pose a threat regarding the infection of animal handlers, veterinarians, and consumers of non-cooked swine meat and pork products. Future studies should address HEV molecular epidemiology and explore the genetic variability and potential transmission among human and animal populations in Brazil.
