# Supplementary material for: Evolutionary study of potentially zoonotic hepatitis E virus genotype 3 from swine in Northeast Brazil
Source: Mem Inst Oswaldo Cruz. 2019 Jun 3;114:e180585. doi: 10.1590/0074-02760180585 (PMC6547807; doi:10.1590/0074-02760180585)
Supplement: Supplementary file 1 [file 1678-8060-mioc-114-e180585-s.pdf]

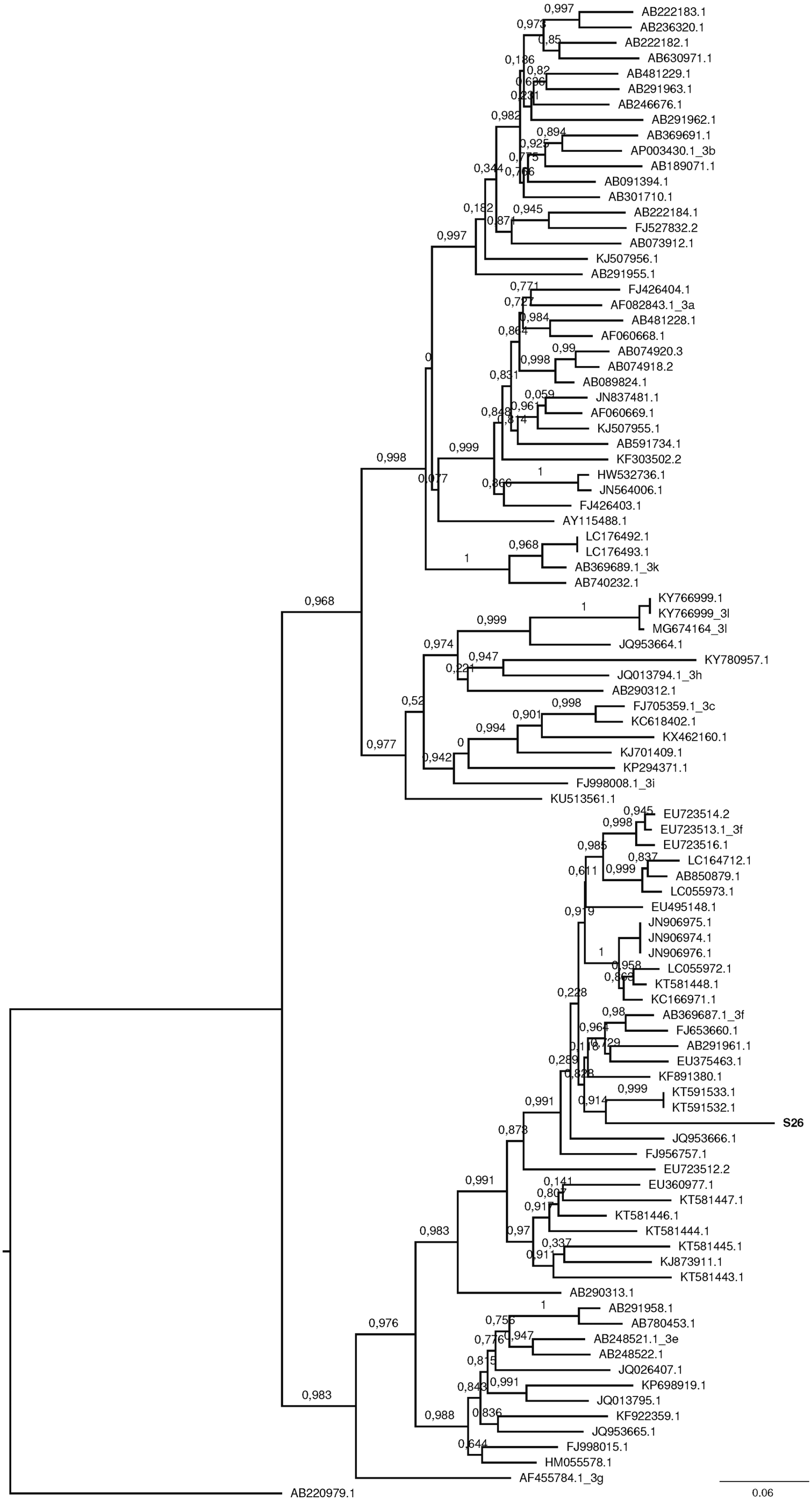

Fig. 1: phylogenetic analyses based on 1503 nt of the hepatitis E virus 3 (HEV-3) capsid gene. Maximum likelihood (ML) phylogenetic tree showing the HEV samples in this study clustering with subtype 3f isolated from humans (A-B). Branch values represent Shimodaira-Hasegawa (SH)-like support values.

CC BY

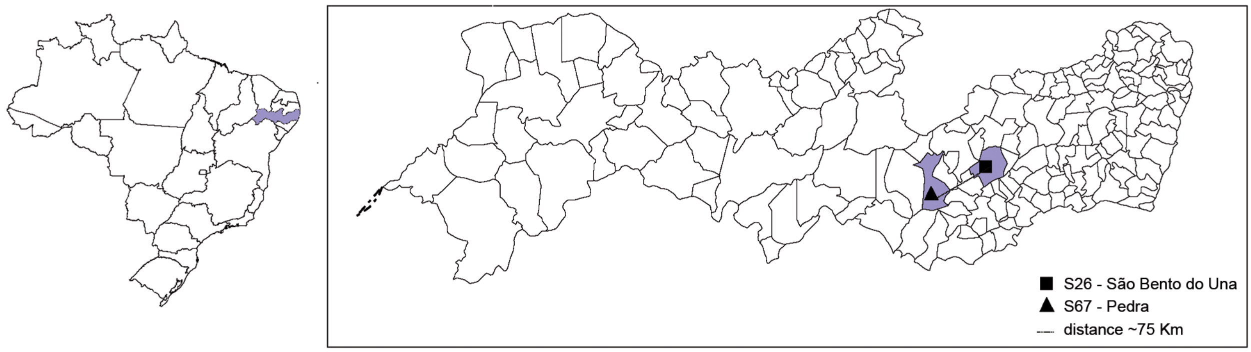

Fig. 2: map showing the locations of the positive animals.
